# Stress and Diet Quality Among Ecuadorian Adults During the COVID-19 Pandemic. A Cross-Sectional Study

**DOI:** 10.3389/fnut.2022.924076

**Published:** 2022-07-07

**Authors:** Victoria Abril-Ulloa, Sueny Paloma Lima-dos Santos, Yadira Alejandra Morejón-Terán, Tannia Valeria Carpio-Arias, Ana Cristina Espinoza-Fajardo, María Fernanda Vinueza-Veloz

**Affiliations:** ^1^Research Group “Public Health, Food and Physical Activity in the Life Cycle”, Nutrition and Dietetics, Faculty of Medical Sciences, University of Cuenca, Cuenca, Ecuador; ^2^Department of Nursing, Ohio University College of Health Sciences and Professions, Athens, OH, United States; ^3^Research Program Social Change, Asthma and Allergy in Latin America – SCAALA, Federal University of Bahia, Salvador, Brazil; ^4^Research Group on Food and Human Nutrition (GIANH), Escuela Superior Politécnica de Chimborazo, Riobamba, Ecuador; ^5^Department of Community Medicine and Global Health, Institute of Health and Society, Faculty of Medicine, University of Oslo, Oslo, Norway; ^6^Department of Neuroscience, Erasmus Medical Center, Rotterdam, Netherlands

**Keywords:** COVID-19 pandemic, stress, diet quality, palatable foods, Ecuadorian adults

## Abstract

**Background:**

Stress has been associated with food habits. Stress changes eating patterns and the salience and consumption of hyperpalatable foods. During the lock-down due to the COVID-19 pandemic, stress was very common.

**Objective:**

We investigated the association between stress and diet quality in Ecuadorian adults during the COVID-19 pandemic.

**Design:**

A cross-sectional study.

**Setting:**

Data was collected using a self-administered online survey. Stress was measured using the Perceived Stress Scale (PSS-14), and diet quality was evaluated using the Global Diet Index (GDI). A linear regression model with restrictive cubic splines was used to investigate the association between stress and diet quality.

**Participants:**

Participants were recruited by convenience sampling, including a total of 2602 individuals. Most participants were female (68.57%) and had university education (78.52%), with a median age of 25 (IQR: 25, 37).

**Results:**

Stress was reported by 26.06% of participants. The majority of individuals (75.79%) reported having a diet that needed changes or an unhealthy diet. Independently from biological sex, age, level of education, people/room ratio, economic allowance, and expenses for food, stress was statistically significantly associated with diet quality (*p* = 0.035). The association between stress and diet quality was inverse and non-linear; higher stress levels were associated with poorer diet quality. The consumption of palatable foods was not statistically significant associated with stress.

**Conclusions:**

Stress is associated with poorer diet quality. Public health measures to improve the mental health and lifestyle of the population are needed during the lock-down of the pandemic.

## Introduction

At the end of 2019, the Coronavirus pandemic begins. In January 2020, the World Health Organization declared a health emergency due to the high number of infected people and the high death rate due to the disease caused by the coronavirus (COVID-19) (1, 2). This situation has had an impact on the family and world economy, on people's lifestyles and mental health, with reports of stress, changes in eating habits, and physical activity in several studies (3). Eating well helps maintain health and physical activity (4), thus improving the enjoyment of life. Good diets and eating habits are critical for proper growth and development and disease prevention. Some different health problems are caused by poor diet and nutrition (5).

Most humans experience altered eating behaviors under stress, with approximately 40% eating more and 40% eating less than usual. In addition, under stress, most people report increased intake of highly palatable foods, regardless of hyperphagia or hypophagia. The consumption of processed products is related to overweight and obesity in all age groups, leading to the development of chronic non-communicable diseases that are a severe public health problem (3, 6).

Unhealthy eating habits are related to stress, anxiety, and depression (7). Some studies show the relationship between the consumption of processed foods and simple carbohydrates with psychological problems, including depression (8, 9). For example, acute stress is associated with loss of appetite, resulting in insufficient consumption. In contrast, chronic stress is associated with a greater preference for appetizing foods rich in sugar and fat (10).

Regarding the quality of the diet, some studies have found a significant relationship between the quality of the diet and the presence of stress. And other studies in which this association has not been found (7, 11). Park et al. (12), showed changes in eating habits in 1 out of 2 adults, they reported consuming more unhealthy snacks and desserts during the initial phase of the COVID-19 pandemic. Consumption of these less healthy foods was higher in younger adults. Dietary changes to less healthy foods can affect long-term metabolic health. In general, during this time of the COVID-19 pandemic, the quality of the diet has decreased, and psychological illnesses, such as stress, anxiety, and depression, have increased. To improve these psychological illnesses, consuming foods rich in omega 3 and reducing the consumption of simple sugars and saturated fats are recommended (13, 14). This research aimed to analyze the relationship between perceived stress and the global quality index of food.

## Methodology

### Design and Context

The present work is an observational cross-sectional study. It is part of a project called EFRICA, currently being carried out in Ecuador. Data collection took place between January and February 2020. At that time, confinement in Ecuador was mandatory. To collect the data, a self-administered survey was used.

### Sample

Individuals of both genders over 18 years old were invited to participate in the study. A non-probabilistic convenience sampling was implemented to recruit participants. Participation invitations were sent through different platforms and social networks, including Facebook, Instagram, Twitter, and WhatsApp. Participation is voluntary and self-selected. In total, 2602 adults voluntarily agreed to participate.

### The Survey

The online self-administered survey was reviewed by four health and nutrition experts participating in the project EFRICA. The survey was first applied to 30 Ecuadorian adults (15 men and 15 women, between 18 and 64 years old) to check for errors and inconsistencies. After corrections were implemented, the survey was distributed through social networks using Google Forms. The survey had four sections: Section 1 included a presentation, objectives, and an informed consent form; Section 2 was meant to collect socio-demographic data; Section 3 included questions regarding diet quality, and Section 4 had questions to assess perceived stress. Diet quality and perceived stress were evaluated using validated questionnaires (15).

### Variables

#### Diet Quality

The global diet index was used to measure the quality of the diet. This instrument contains 12 variables, which include the frequency of daily or weekly consumption of group (a) 5 groups of healthy foods: fruits, vegetables, fish, legumes, and milk or derivatives; group (b) 4 unhealthy foods: fried foods, sugary drinks, cakes/cookies/pastries/sweets and sugar, and group (c) main meals: breakfast, lunch, and dinner.

For each of these variables, the following alternatives were considered: 2 or more times a day, 1 time a day, 4–6 times a week, 2–3 times a week, once a week, and occasionally or never. Based on this information, scores were assigned, with 10 being the ideal value and a score of 1 being the least healthy for each index parameter. The maximum score with the 12 parameters studied was 120, classified according to the following criteria: Healthy eating: 90–120 points; a diet that needs changes: 60–89 points; unhealthy eating: <60 points.

#### Stress

The experience of stress was assessed using the perceived stress scale (PSS), which evaluates the degree to which situations in one's life are experienced as stressful (15). The PSS consists of 14 questions with five possible answers and scores: never = 0, hardly ever = 1, occasionally = 2, ofte*n* = 3, always = 4, and items, each of which respondents are ranked from 0 (never) to 4 (very often). Questions 4, 5, 6, 7, 9, and 10 are “positive” and therefore scored inversely. PSS-14 scores range from 0 to 56. The higher the overall score, the higher the level of perceived stress. A cut-point corresponding to the group's median was established for group participants as having or not experiencing stress.

#### Statistical Analysis

Categorical variables were summarized by frequency and percentages. Median and interquartile ranges (IQR) were used to summarize numerical variables since all were non/normally distributed. Association between categorical variables and stress was tested using the Chi^2^ test. Association between numerical variables and stress was tested using the rank-sum test. The association between diet quality and stress was analyzed by implementing a linear regression model with restrictive cubic splines (RCS) to model non-linearity. In this model, the score of diet quality (GDI) was included as the outcome variable and that of Stress (PSS) as an explanatory variable. The model was adjusted by age (male, female), age (years), level of education (No education/primary/secondary, university), economic allowance (yes, no), and expenses per month in food (< $400, $400–$800, >$800). The RCS regression model determined the shape of the relationship between diet quality and stress without any prior assumption. RCSs fitted a smooth continuous curve of adjusted means with 95% confidence intervals (95% CIs) across PEE levels. RCSs allowed for changes in the function at defined knot points and restricted the splines to linear relationships at the tail ends. Knot points were located at percentiles 5, 27.5, 50, 72.5, and 95 altitudes, as previously recommended to avoid forcing curvature or inflections (16). R version 3.6.3 (2020-02-29) and related packages, including rms were used to analyze the data (16, 17).

## Results

### Sample Characterization

The sample included 2602 adults, of whom 1776 (68.57%) were female and 814 (31.43%) were male, with a median age of 25 (IQR: 25, 37). Most participants had a university education (*n* = 2043, 78.52%). They did not receive any economic allowance from the government to cover their expenses (*n* = 2463, 96.32%). The median ratio between the number of people living at home and the number of rooms was 1.4 (IQR: 1, 2). From the participants 48.82% (*n* = 1059) reported spending < $400 per month, 47.30% (*n* = 1025) between $400 and $800 per month and 3.87% (*n* = 83) > $800 per month in food. Sample characteristics are summarized by having or not experiencing Stress in Table 1.

**Table 1:** General characteristics of the sample by level of stress.

		**No stress**	**Stress**	**Missing n**	**Test**	***P***
		**(n = 1924, 73.94%)**	**(n = 678, 26.06%)**	**(%)**		***value***
**Biological sex**	Female	1210 (63.22)	566 (83.73)	12 (0.00)	Chisq. (1 df) = 97.51	<0.001
	Male	704 (36.78)	110 (16.27)			
**Age (years)**	Median (IQR)	26 (21.00, 38.00)	23 (20.00, 30.00)	22 (0.84)	Ranksum test	<0.001
**Level of education**	No	392 (20.37)	167 (24.63)	0 (0.00)	Chisq. (1 df) = 5.39	0.020
	education/primary/	1532 (79.63)	511 (75.37)			
	secondary University
**Overcrowding**	Median(IQR)	1.33 (1.00, 2.00)	1.5 (1.00, 2.00)	44 (1.69)	Ranksum test	<0.001
**Economical allowance**	No	1816 (96.19)	647 (96.71)	45 (1.73)	Chisq. (1 df) = 0.39	0.535
	Sí	72 (3.81)	22 (3.29)			
**Expenses per month**	< 1 basic salary	796 (49.08)	263 (48.08)	433 (16.64)	Chisq. (2 df) = 4.00	0.136
	1-3 basic salaries	756 (46.61)	270 (49.36)			
	> 3 basic salaries	70 (4.32)	14 (2.56)			
**Diet quality**	Median (IQR)	77.5 (66.50, 90.00)	73.75 (60.50, 85.88)	0 (0.00)	Ranksum test	<0.001
	Healthy	498 (25.88)	132 (19.47)		Chisq. (2 df) = 33.41	<0.001
	Need changes	1164 (60.50)	394 (58.11)			
	Not healthy	262 (13.62)	152 (22.42)			

*Sample included 2602 adults. Having or not experiences stress was measured using the perceived stress scale. Diet quality was measured using the global diet index. Symbology and abbreviations: IQR, interquartil range; GDI, general diet index; df, degrees of freedom; n, number; %, percentage*.

The median score of PSS was 18 (IQR: 15, 22), and 26.01% (*n* = 678) reported having experienced stress. The median score of GDI was 76.50 (IQR: 65.00, 88.50). The majority of participants (*n* = 1972, 75.79%) reported having a poor diet quality [needed changes, *n* = 1,558 (59.88%); unhealthy, *n* = 414 (15.91%)]. It was observed that those who reported having experienced stress also reported a worse diet quality (Table 1).

### Association Between Stress and Diet Quality

**Figure 1:**
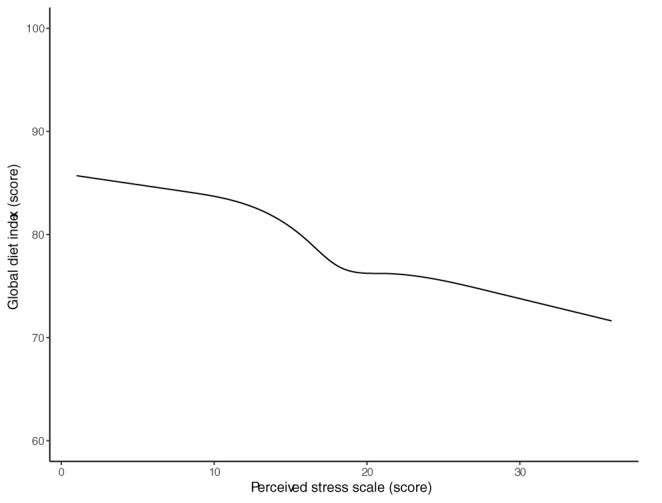
Association between diet quality and stress. Predicted means of GDI and respective 95% confidence intervals are shown for different levels of stress. An inverse nonlinear association between diet quality and stress is shown in that, a worse diet quality is associated with higher levels of stress. Data was modeled using linear regression with resctrictive cubic splines (see Methods section).

To study the association between stress and diet quality, we implemented a linear regression model with RCS (see Methods). We found that independently of biological sex, age, level of education, the ratio of people/room, economic allowance, and expenses in food, stress was statistically significantly associated with diet quality (*F* = 2.88, df = 3, *p* = 0.035). The association between stress and diet quality was inverse and non-linear. That is, higher levels of stress (measured by PSS) were associated with lower diet quality (measured by GDI) (Figure 1). Predicted GDI means for different PSS values are shown in Table 2.

**Table 2:** Predicted means of GDI.

**Variable**		**Mean**	**LCI**	**UCI**
**PES**	1Q (15)	80.72	78.77	82.66
	Median (18)	77.01	75.05	78.98
	3Q (22)	76.16	74.22	78.10
**Biological sex**	Female	77.01	75.05	78.98
	Male	74.12	71.98	76.26
**Age (years)**	1Q (18)	76.49	74.44	78.53
	Median (25)	77.01	75.05	78.98
	3Q (37)	77.92	75.88	79.96
**Ratio person/room**	1Q (1)	79.51	77.49	81.54
	Median (1.4)	77.01	75.05	78.98
	3Q (6)	70.75	63.97	77.54
**Economical allowance**	No	77.01	75.05	78.98
	Yes	73.25	69.24	77.27

*Adjusted predicted means and their confidence intervals are showed for stress (quartiles), sex, age (quartiles), ratio number of persons/room (quartiles), economical allowance. Symbology and abbreviations: 1Q, first quartil; 3Q, third quartil; LCI, lower 95% confidence interval; UCI, upper 95% confidence interval*.

Diet quality was also significantly associated with age (*F* = 5.65, df = 1, *p* = 0.018), biological sex (*F* = 13.64, df = 1, *p* < 0.001), rario people/room (*F* = 3.83, df = 3, *p* = 0.001), and economical allowance (*F* = 4.06, df = 1, *p* = 0.044), but not to level of education (*F* = 0.85, df = 1, *p* = 0.357) or expenses in food (*F* = 0.95, df = 2, *p* = 0.388). In this way, a worse quality of diet was evident in young compared to old people, in males compared with females, in people living in overcrowding, and among those receiving an economical allowance from the government. Predicted means of GDI for significantly associated covariates are shown in Table 2.

### Palatable Foods and Stress

There was not a statistically significant association between different palatable foods and stress, with the exception of consumption of cookies (Supplementary Table 1). In this way, a higher consumption of cokies was associated with 0.013 points reduction on the stress score.

## Discussion

### Stress and Lockdown

People's mental health is severely affected during pandemics, and this has already been observed, for example, in the SARS pandemic, where it was observed that episodes of anxiety (18) and stress (19) were common among people.

The increase in mental health problems in the COVID-19 pandemic has been evidenced in numerous scientific studies, including several meta-analyses (20, 21). In this sense, it has been observed that the highest risk groups are health professionals (22), women, young adults, and people with low incomes (23, 24). Moreover, the World Health Organization (25) has issued public interest guidelines to address psychological problems that may arise in times of pandemics.

Our data indicates that 26.01% of people experienced stress during the pandemic. Studies on the Ecuadorian population have also shown an increase in stress in the population (24) and a relationship between stress and increased depression (26). Statistics alert public health systems to create emerging policies supported by health professionals to generate actions to protect mental health (27). However, the actual consequences in the medium and long term of increasing mental health problems in the population are a projection and should continue to be studied.

### Diet Quality and Lockdown

People are social beings, and confinement has had several consequences. Staying home for days with little or no contact with other people has caused some people to overeat or more frequently as a mechanism to reduce fear and anxiety (28). In addition, limited access to groceries led many people to have little access to fresh foods such as fruits and vegetables. The most convenient for them was to buy processed products, junk food, and ready-to-eat cereals rich in fat, sugar, and salt (29). This situation could lead to an increase in sweets, fats, and alcohol (30), salty snacks, and fast foods every day that is very dangerous for the health (31).

Our study found that 75.79% of the participants had a poor-quality diet. It could be due to little access to fresh food and money available for food during the month. It has been reported that the socioeconomic level influences the quality of food, finding that at the low socioeconomic levels, the consumption of fruits and vegetables is lower (32). On the other hand, social determinants such as loss of employment or few job opportunities and lack of access to recreational activities due to confinement impacted the diet quality, negatively affecting the most vulnerable groups (33).

These findings may be in contrast to what has been found in other studies where it has been found that during confinement, the consumption of fruits, vegetables, and organic foods improved, which may be related to cultural and economic factors (29, 33). It is also necessary to consider that food insecurity and food quality before the COVID-19 pandemic could worsen during confinement, affecting the most vulnerable groups (33).

It is necessary to carry out studies that allow knowing the determining factors of these changes in eating habits in the Ecuadorian population to promote an accessible and sustainable healthy diet. On the other hand, almost all the studies during confinement were carried out online. It would be essential to know how people's eating habits with little or no access to the internet changed because these groups possibly show other consumption patterns.

### Diet Quality and Stress

Stressful situations stemming from the COVID-19 pandemic have been linked to unhealthy habits (34). One of the concerns of health professionals corresponds to the unhealthy changes that the stressed population can have in their diet (34).

Stress can lead to emotional overeating and high energy food craving (35). In general, bad eating habits are related to the presence of stress (7). In this sense, there has been an increase in palatable foods and alcohol consumption and a decrease in the consumption of fruit and vegetables in various countries (36). Also, in studies, a relationship has been found between the consumption of simple meat hydrates and processed foods with psychological problems (7). It should be mentioned that there is no single validated index that allows measuring the quality of the diet in all countries. However, our work will give us a global perspective on diet quality in the study subjects (37). Our results show a relationship between increased stress, unhealthy diet, and health problems. Similar findings show that mental health is directly related to food consumption, so it must be considered in dietary prescriptions and public health policies (38).

Prolonged stress causes the release of cortisol, which increases the feeling of appetite. It has been reported that stress due to the COVID 19 pandemic has been able to influence the increase in food consumption (52% of people), including energy-rich products and snacks after dinner, which in turn could have an impact on the increase in weight that is a risk for obesity; it affects people's health and well-being (35, 39).

The decrease in the consumption of fruits, vegetables, and a variety of foods, can also impact the immune system, making it more sensitive to infections and diseases. Therefore, ensuring adequate intake of vitamins, minerals, and micronutrients through food is essential to strengthen the immune system and maintain good health (40). In addition to promoting healthy eating, it is essential to promote mental health care as a priority for the population's well-being, even more so with the evidence that stress has a negative impact on people's physical and mental health (41).

### Strengths and Limitations of the Study

This study had some limitations. First, a virtual survey was used to collect the information that, although true, allowed us to obtain the data in a relatively short time. Second, we work with a non-representative sample, so we cannot determine the generalizability of the results. Third, it is possible that as a cross-sectional virtual survey self-reported survey data, the results potentially suffer from recall and reporting bias. Fourth, we could not evaluate food and beverage consumption before the COVID-19 outbreak. Fifth, although we tried to reach more male participants, sex distribution is not balanced. Also, we do not consider BMI for the adjustment because the body image was reported. The strength of this study is that, to our knowledge, this is the first study to evaluate stress.

Although this study recognizes some limitations, including the lower participation of men compared to women, it is important to indicate that the number of participants at the national level is relatively high and allows the identification of cardiovascular risk factors that can have a strong impact on the Ecuadorian population. Nevertheless, factors such as stress and diet may differ between genders; the information obtained is the first report in the country and serves as the basis for the formulation of new studies focused not only on identifying these and other cardiovascular risk factors at the population level but also promoting healthy lifestyles and improving quality of life. All of these actions are in order to avoid negative impacts on the health of the Ecuadorian population in the medium and long term.

### Perspectives and Future Studies

They are finding in this study the relationship between food and stress. The diet of these people should be taken into account. New studies should also be done on diet and psychological illnesses.

It could be interesting to analyze these factors in different socioeconomic strata to have a better understanding of the variability o similarities that could show people and in the future design interventions with a focus on public health considering several determinants that could affect the health.

## Conclusions

Our results suggest that higher stress levels (measured by PSS) were associated with lower diet quality (measured by GDI). The consumption of palatable foods associated with stress was not statistically significant. Recent research has analyzed possible associations to stress-related eating. Although the data suggest potentially addictive properties of hyper-palatable foods, there is a debate about food addiction. The lockdown is an excellent strategy to stop the spread of the virus. However, it is essential to consider that physical inactivity, weight gain, behavioral food changes, and social isolation exist during this process, depending on the intensity of stress and environmental factors. By describing several substances involved in appetite regulation and weight control, identifying all centers involved in eating behavior demonstrates the complexity of studying this phenomenon and, consequently, energy homeostasis. Understanding the associations and interactions between stress and diet is essential in developing effective prevention.

## Data Availability Statement

The datasets generated during and/or analyzed during the current study are available from the corresponding author on reasonable request.

## Ethics Statement

The present study has been approved by the University of Cuenca Bioethical Committee (UC-COBIAS-2020-078) and Research Institute (IDI) of Escuela Superior Politécnica de Chimborazo. All participants signed a written informed consent before being considered in the study.

## Author Contributions

VA-U, SS, YM-T, and MV-V: conception and design, analysis and interpretation of data, and drafting of the article. VA-U, SS, MV-V, YM-T, and AE-F: final approval of the version to be published. AE-F: writing. MV-V and AE-F: critical revision for important intellectual content. All authors contributed to the article and approved the submitted version.

## Funding

This study is conducted with the financial support of the Escuela Superior Politécnica de Chimborazo and carried out by the University of Cuenca, State University of Milagro, and the Pontifical Catholic University of Quito. This publication is part of the study Cardiometabolic risk study in Ecuadorian adults. EFRICA-EC.

## Conflict of Interest

The authors declare that the research was conducted in the absence of any commercial or financial relationships that could be construed as a potential conflict of interest.

## Publisher's Note

All claims expressed in this article are solely those of the authors and do not necessarily represent those of their affiliated organizations, or those of the publisher, the editors and the reviewers. Any product that may be evaluated in this article, or claim that may be made by its manufacturer, is not guaranteed or endorsed by the publisher.
