# Supplementary material for: Stress and Diet Quality Among Ecuadorian Adults During the COVID-19 Pandemic. A Cross-Sectional Study
Source: Front Nutr. 2022 Jul 7;9:924076. doi: 10.3389/fnut.2022.924076 (PMC9301032; doi:10.3389/fnut.2022.924076)
Supplement: Supplementary file 1 [file Table_1.pdf]

frontiers

Supplementary table 1. Crude and adjusted models examining the pathways from perceived stress and palatable foods.

Variable	Crude model		Adjusted model*	
	Coefficient	IC95%	Coefficient	IC95%
Cookies	-0.011	-0.023; 0.001	-0.013	-0.025; -0.001
Sugar	-0.009	-0.020; 0.003	-0.008	-0.020; 0.003
Drinks	-0.009	-0.020; 0.003	-0.008	-0.020; 0.003
Fried foods	0.001	-0.002; 0.021	0.010	-0.001; 0.022

*Adjusted for gender, age, level of education, economical allowance and expenses per month in food (see Methods section).
